# A Novel Approach for Determining Cancer Genomic Breakpoints in the Presence of Normal DNA

**DOI:** 10.1371/journal.pone.0000380

**Published:** 2007-04-18

**Authors:** Yu-Tsueng Liu, Dennis A. Carson

**Affiliations:** Moores UCSD Cancer Center, University of California San Diego, La Jolla, California, United States of America; National Cancer Institute, United States of America

## Abstract

*CDKN2A* (encodes p16^INK4A^ and p14^ARF^) deletion, which results in both Rb and p53 inactivation, is the most common chromosomal anomaly in human cancers. To precisely map the deletion breakpoints is important to understanding the molecular mechanism of genomic rearrangement and may also be useful for clinical applications. However, current methods for determining the breakpoint are either of low resolution or require the isolation of relatively pure cancer cells, which can be difficult for clinical samples that are typically contaminated with various amounts of normal host cells. To overcome this hurdle, we have developed a novel approach, designated Primer Approximation Multiplex PCR (PAMP), for enriching breakpoint sequences followed by genomic tiling array hybridization to locate the breakpoints. In a series of proof-of-concept experiments, we were able to identify cancer-derived *CDKN2A* genomic breakpoints when more than 99.9% of wild type genome was present in a model system. This design can be scaled up with bioinformatics support and can be applied to validate other candidate cancer-associated loci that are revealed by other more systemic but lower throughput assays.

## Introduction

Tumors evolve through the continuous accumulation and selection of randomly mutated genes. While sets of advantageous mutations are selected in tumors, neutral or even slightly detrimental mutations may also occur due to genomic instability and genetic drift. Recently, much effort has been expended to identify in primary human cancers point mutations in the exons of cancer-related genes. However, systemic mapping of genomic DNA rearrangements has lagged behind, due to technical difficulties in detecting smaller deletions, tumor heterogeneity, and the necessity to purify malignant from normal cells [1]. Historically, such work was done by time consuming and labor intensive genetics and molecular cloning on established cancer cell lines [2], [3], [4]. One of the most striking examples is the homozygous deletion of the *CDKN2A* (*INK4A/ARF*) tumor suppressor locus, which was discovered in this and other laboratories [3], [4], [5], [6], [7], [8]. The *CDKN2A* deletions occur early during tumor development [9], [10], [11]. The p16^INK4a^ (one of the *CDKN2A* products [12]) protein constrains cell cycle progression by the Rb pathway and may be responsible for the decline in the replicative potential of stem cells during aging [13]. The p14^ARF^ (the other alternative reading frame of *CDKN2A* [14]) gene product regulates the expression of MDM2, the turnover of p53, and thereby controls the cellular response to stress (reviewed in [6], [7], [8], [15], [16], [17]). Because the Rb and p53 pathways are central to cancer gate-keeping and caretaking [18], [19], strong selection pressures exist for the disruption of the entire *CDKN2A* gene segment on both chromosomes. Few other deletions are as well characterized, although it is expected that more will be found when more data from array based comparative genomic hybridization (array-CGH) are reported and also through The Cancer Genome Atlas (TCGA) project [20], [21], [22], [23], [24]. It will be important to validate the relevance of those genomic rearrangements to cancer development since many of the genomic structural changes may be simply due to genome instability in cancer. Large scale studies with clinical samples will be the most reliable confirmation.

While point mutations and very small insertions or deletions in genomic DNA can be detected by exon re-sequencing, it can be more difficult to detect gene dosage changes of larger genomic fragments, especially deletions [1]. Current established techniques for deletion mapping, including Southern blotting [25], fluorescent *in situ* hybridization (FISH) [26], quantitative PCR [26], [27], [28], [29], [30], and array-CGH [31] rely on the absence of a detectable wild type signal [1]. This is problematic when a significant number of normal cells are present in a tumor sample. Array-CGH has the potential to analyze alterations of DNA copy number on a genome-wide scale with relatively high resolution, depending on whether BACs, PCR products or oligonucleotides are used for the array elements. However, these techniques often fail where there is a heterogeneous cell population or samples of poor quality [31]. FISH is less vulnerable to the presence of heterogeneous cell populations, but has relatively low resolution and is difficult to scale up. Except for FISH, the other techniques mentioned are not practical for mapping genomic translocations and inversions. End-sequencing profiling was developed to address this issue but the approach was costly and hard to scale up [32]. Therefore, there is a need to develop a scalable approach for detecting such genomic structural changes in solid tumors where heterogeneous cell populations are present.

Here we report a novel approach, designated as Primer Approximation Multiplex PCR (PAMP), to enrich small amounts of deleted genomic DNA sequences in the presence of wild type DNA. The genomic locations of the enriched sequences are subsequently decoded by a genomic tiling array and confirmed by sequencing.

## Results

### *CDKN2A* locus

**Figure 1:**
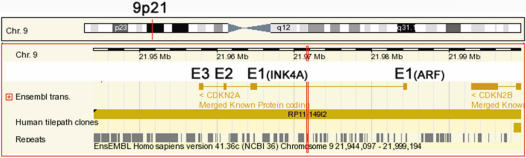
The *CDKN2A* locus. The genomic map covers about 55 kb around *CDKN2A* according to Ensemble [59]. *CDKN2A*/B is located at chromosome 9p21 and their RNA products are encoded by the reverse strand. *CDKN2A* encodes 2 proteins (p16^INK4A^ and p14^ARF^) that share the same exons 2 and 3. The first exons of *INK4A* and *ARF* are about 20 kb apart. *CDKN2B* encodes p15^INK4B^ that is homologous to p16^INK4A^. In addition to transcripts, the map also shows repetitive sequences (Repeat) and available BAC clone (Human tilepath clones), RP11-149I2.

The *CDKN2A* is located on chromosome 9p21 (Figure 1). It encodes two proteins in different reading frames: p16^INK4A^ and p14^ARF^, which both have 3 exons and share exons 2 and 3. *CDKN2B* (p15^INK4B^) and *MTAP* (methylthioadenosine phosphorylase) (not shown) are centromeric and telomeric neighboring genes respectively [3], [17], [33], [34]. BAC clone RP11-149I2 contains the whole *CDKN2A* genomic fragment and was used as template to generate probes (excluding repetitive regions) for printing on the minigenomic tiling array. The frequency of repetitive sequences predicted by RepeatMasker is shown at the bottom of the diagram.

### Primer Approximation Multiplex PCR (PAMP)

**Figure 2:**
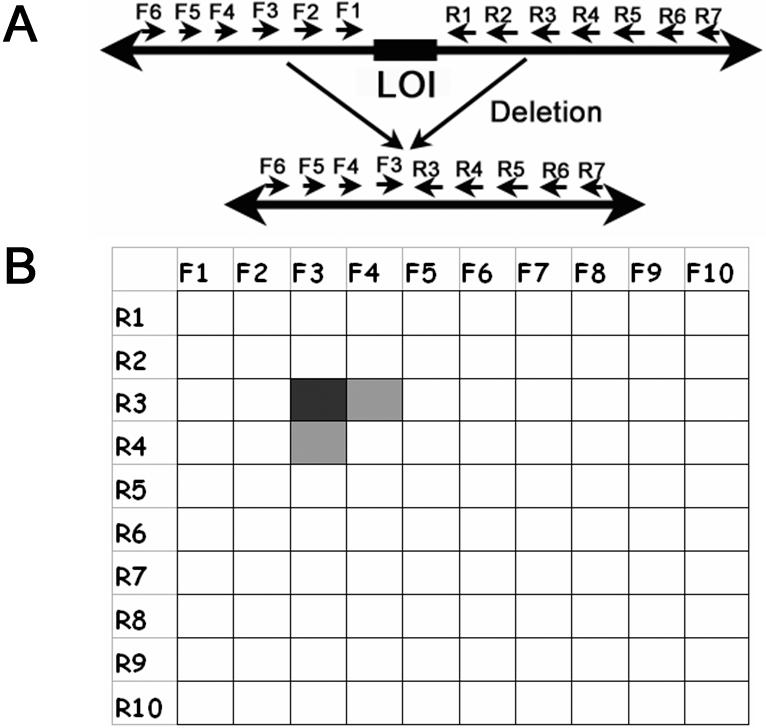
Primer Approximation Multiplex PCR (PAMP). The efficiency of PCR amplification is inversely related to the distance of upstream and downstream primers. In this example, primers to amplify genomic sequences around the locus of interest (LOI) are divided into 20 groups: 10 each for forward (F1–F10) and reverse (R1-R10) groups (A). While all of the possible forward and reverse primer pairs are too far to each other for PCR amplification in wild type genome, certain pair of primers is brought closer (“approximated”) due to deletion (F3 and R3) in mutated genome. Multiplex PCR reactions are set and represented as a matrix to include one forward and one reverse primer group. The expected PCR results are shown as gray scale shadows in the matrix (B). This example shows that only group pairs close to breakpoint give PCR products (F3-R3, F3-R4, F4-R3).

It is difficult to detect a small fraction of deleted mutant genomic DNA in the presence of a vast excess of wild type DNA with array CGH or other popular molecular biology tools [26], [27], [35]. In typically contaminated tumor samples, genomic DNA is composed of various ratios of WT and *CDKN2A* deficient DNA. We aimed to take advantage of the fact that a shorter deleted genome sequence should be preferentially amplified compared to a much longer WT sequence using “approximated” flanking primers (Figure 2A) [36].

The approach is illustrated in Figure 2. In this example, relatively even-spaced primers (average 1 kb apart) surrounding the locus of interest are divided into 20 groups for PCR. There are 10 groups each of forward primers, F1, F2 …, F10 and reverse primer R1, R2, …,R10, respectively (Figure 2A). Therefore, there are 100 pairs (F1-R1, F1-R2, …, F1-R10; F2-R1, F2-R2,…, F2-R10; …; F10-R1, F10-R2,…., F10-R10) of PCR reactions (Figure 2B). It is expected that only one or two pairs of PCR reactions will produce specific PCR products spanning the deletion boundary, since the other primer pairs should be too far from the breakpoint for efficient amplification. Then aliquots from each reaction can be mixed to hybridize on a single genomic tiling array. Unlike traditional array-CGH, it is expected that only spots representing genomic sequences near the breakpoints will be theoretically lit up, which was confirmed in the following experiments.

In order to increase the throughput and reduce the cost of reagents, every forward (F1-10) and reverse (R1-10) primer group can have multiple primers. Therefore, every PCR group (for example F1-R1) pair becomes multiplex PCR. Therefore, we designated this procedure as Primer Approximation Multiplex PCR (PAMP).

### Deletion breakpoint cloning by PAMP and minigenomic tiling array

**Figure 3:**
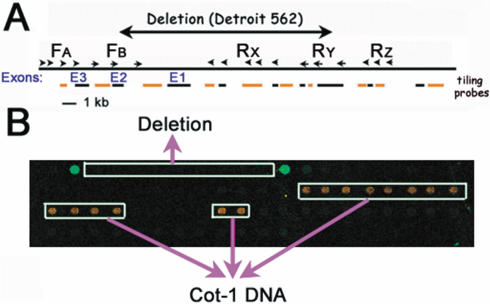
Breakpoint identification by PAMP with an *INK4A* minigenomic tiling array. (A) Five groups of primers (F_A_, F_B_, R_x_, R_Y_ and R_Z_, the small arrows and arrow heads) near the potential breakpoints were generated for PAMP based on our previous mapping [3]. The mapped *CDKN2A* breakpoints of the Detroit 562 cell line (Figure 5) are indicated for clarification. The “E1”, “E2” and “E3” designations (blue fonts) are the relative positions of *INK4A* exons. The first exon of *ARF* is further to the right of this diagram and is not covered by this array. The tiling probes for the array are indicated with two alternating colors (short black and orange lines) for ease of identification. (B) The first row of the *INK4A* minigenomic array was spotted with the tiling probes shown in panel A. Cot-1 DNA (repetitive sequence of genomic DNA) spots are indicated on this array. The rest of the spots are herring sperm DNA. Both Cot-1 and herring sperm DNA are used as nonspecific controls. This array was hybridized with labeled samples derived from two cell lines. The same sets of primers (F_A_, F_B_, R_Y_ and R_Z_) were used for PAMP reactions on Detroit 562 (mutant) and HEK293 (wild type) genomic DNA to map the potential *CDKN2A* breakpoints. The amplicons were labeled with different dyes, yielding a green signal (Cy-3) for the mutant sample and a red signal (Cy-5) for the wild type sample, to be simultaneously hybridized on the array (two-color array). The two green spots on the first row revealed the breakpoint location as been discussed in Figure 2.

We reported previously that the Detroit 562 cell line has an approximate 20 kb (including *INK4A* exons 1 and 2) deletion on chromosome 9p21 [3]. We used this cell line to test our deletion scanning approach. Four groups (F_A_, F_B_, R_Y_ and R_Z_) of primers were used for four PAMP reactions (Figure 3A) using genomic DNA template either from Detroit 562 (*CDKN2A* deficient) or HEK293 (*CDKN2A* wild type) cell lines. Aliquots of all 4 PAMP reaction products were pooled and labeled for hybridization on an *INK4A* minigenomic tiling array that covers about 25 kb, including all of the exons of *INK4A*. As predicted in Figure 2, only spots with probes close to the breakpoints hybridized to the amplicons when Detroit 562 genomic DNA was used as a template (Figure 3B). Almost no signal was detected when HEK293 genomic DNA was used as a template. The control HEK293 sample had a significantly higher signal on Cot-1 DNA spots despite its general absolute signal intensity is low.

**Figure 4:**
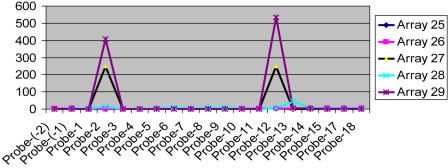
Pooling of individual PAMP reactions for single array hybridization. Four groups (F_A_, F_B_, R_Y_ and R_Z_) of primers were used for four PAMP reactions each by pairing all of the possible forward and reverse primer groups using Detroit 562 (mutant) and HEK293 (control) as templates. The procedure has been briefly described in Figure 3. The products were labeled and used for array hybridization: F_A_-R_Y_ for array 25; F_A_-R_Z_ for array 26; F_B_-R_Y_ for array 27 and F_A_-R_Z_ for array 28. Aliquots of the individual PAMP samples were also pooled together and labeled for array hybridization (array 29, its array image is shown in Figure 3B). The results are presented with ratio signal intensity (Y-axis) of samples from Detroit 562 (mutant) and HEK293 (control) against the probe location (X-axis). The breakpoints can be identified through this plot by finding the two peaks that are analogous to the bright green spots in Figure 3B.

In addition, four separate arrays were used to hybridize the individual PAMP products described above. A simple plot of signal intensity ratio of mutant/WT PCR products on the tiling array revealed the genomic location of the breakpoint (Figure 4). This analysis shows a very straightforward readout—the location of the deletion is bordered by two peaks. Only F_B_-R_Y_ (array 27) and all products pooling (array 29) produce the same result as shown in Figure 3. In contrast, the other three pairs yielded only faint background signals on the arrays. This result indicates that PAMP product pooling with a single array analysis gives the same breakpoint information as four individual arrays. The data support the original experimental predictions, and suggest that the procedure should be generally applicable for deletion and translocation scanning.

**Figure 5:**
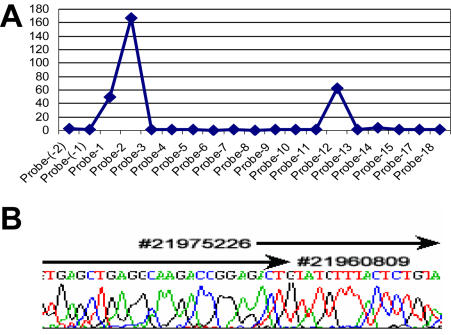
Breakpoint mapping of Detroit 562 cell line. To map the exact breakpoint, a nested set of PCR primers were designed for uniplex PCR based on the previous PAMP results (Figures 3 and 4). The PCR products were used for labeling and hybridized on the array and also for agarose gel electrophoresis. The array data is shown as the same plot in Figure 4. A single major band on the agarose gel was excised and purified for sequencing (B). The breakpoint is indicated (from #21975226 to #21960809 according to NCBI human genome sequence build 36).

In order to pinpoint more precisely the area of deletion, nested PCR with pairs of specific primers was designed according to the earlier PAMP results. The PCR product was labeled for array hybridization, yielding a result very similar to that shown in Figure 4 and is shown in Figure 5A. Furthermore, the single major product of the PCR reactions was resolved by agarose gel electrophoresis, excised, extracted and sequenced (Figure 5B). The breakpoint cloned is in agreement with two other reports (Figure 5B) [37], [38].

To mimic the heterogeneous population of cancer and host cells typically found in solid tumors, various amounts of genomic DNA derived from Detroit 562 (mutant) and HEK293 (wild type) were mixed for PAMP and array hybridization. In order to test the sensitivity of our approach, we performed a titration experiment. The total genomic DNA for each assay was kept constant (100 ng). This is equivalent to about 28,000 copies of haploid genome (based on the estimate of 2.8×10^5^ molecules/µg of haploid genome). The *CDKN2A* deleted cell line Detroit 562 was serially diluted with *CDKN2A* wild type HEK293 as shown in the Table 1. The assay was able to detect approximately 1 breakpoint sequence in the presence of an approximately 2000 fold excess of wild-type genome with sensitivity of 5–16 such molecules (Table 1). Thus, the PAMP approach provides a method for detecting genomic DNA deletions in the presence of more than 99.9% wild type DNA.

**Table 1:** The sensitivity of PAMP assay

Complexity Detroit 562: Total	Absolute genome copy number of Detroit 562	Array Result
1∶1	28000	P
1∶10	2800	P
1∶50	560	P
1∶100	280	P
1∶200	140	P
1∶600	47	P
1∶1800	16	P
1∶5400	5	N
0∶1	0	N

Total input of genomic DNA is 100 ng for each reaction.

P: positive. N: Negative

**Figure 6:**
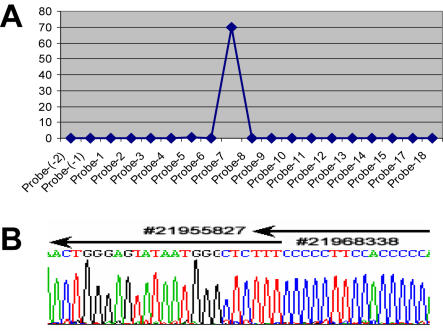
Breakpoint mapping of Hs578T cell line. Two groups of primers: F_A_ (F_A1_-F_A4_) and R_X_ (R_X1_-R_X5_) were used for PAMP based on our previous mapping. The product was labeled for array hybridization (A). Only single peak is evident from the plot. It indicates the location of the other breakpoint is not covered by this minigenomic array. Two primers (R_X3_ and R_X4_) located near the genomic location of the probe (human chromosome 9, 21969229 to 21970477, NCBI build 36) that was hybridized and two primers (F_A1_ and F_A2_) located outside the array coverage were chosen for uniplex PCR. The F_A2_-R_X3_ pair is expected to have the shortest distance when a deletion occurs. A band of about 2 kb on agarose gel was excised from the gel, purified and sequenced (B). The breakpoint and location is indicated (from #21955827 to #21968338 according to NCBI human genome sequence build 36).

The PAMP breakpoint cloning strategy was confirmed in another cell line. Because our array only covers 25 kb of the genome, we scanned our previous mapping information on 100 cell lines to find one that might have breakpoints within this region [3], [33]. The Hs578T breast cancer cell line has a deletion in p16^INK4A^ exons 1-3. With primers within and telomeric (F_A_ and R_X_ groups, Table 2) to the genomic fragment, we performed PAMP and array hybridization, and identified a single spot on the array (shown as a single peak in Figure 6A). The sequence of this probe is located from 69971 to 71219 in the RP11-149I2 BAC sequence (GenBank accession AL449423). Therefore, the centromeric end of the breakpoint should be located near this region. Uniplex PCR with primers from the two groups for PAMP was performed for sequencing. A PCR product about 2 kb was generated with a pair of primers and was subjected to direct sequencing (Figure 6B). The centromeric end of the breakpoint identified by PAMP is consistent with a previous report [37]. The telomeric end of the breakpoint was inferred from the primers used for PAMP and also confirmed by direct sequencing, although there was no genomic probe near the breakpoint that was included on the array.

**Table 2:** Primers for PAMP

Primer	Sequence	Location
**F_A1_**	TTTCTGCTATTTCCTGAAC	55654–55672
**F_A2_**	CAGTATGCGTGTGCTCAG	56279–56296
**F_A3_**	AAATAGAGGTGCAGTGCTC	57913–57931
**F_A4_**	GGGAAGGCATATATCTACG	58800–58818
**F_B1_**	ATTAATTGTGCTTGAAGAGG	60231–60250
**F_B2_**	AGGCCTTGAACTAGCAGAG	61358–61376
**F_B3_**	CCAGGTTTATGATTTGAGAG	63412–63431
**R_X1_**	GAAATGTGTTCCCTCCCTC	68061–68043
**R_X2_**	GGATAATGGACTTCAATTTC	68846–68827
**R_X3_**	CCATCCTCTCTACTCATAAG	70476–70457
**R_X4_**	TCATTAGAAAGGCCATGGAC	71219–71200
**R_X5_**	TAAATTAAAGGGATGCATGG	72297–72278
**R_Y1_**	TAATTATTGCTTTGTGTGGG	74054–74035
**R_Y2_**	ATGATTAAGGATATGGTTGG	74555–74536
**R_Y3_**	GTATTCAGACTCCTGGTATG	76585–76566
**R_Z1_**	TTATGATCCAGACCAGGCTC	78114–78095
**R_Z2_**	TTATCTTTGAATTGAGGTCC	78543–78524
**R_Z3_**	GGGTGGTTGAAGAAATTCTC	79277–79258

The location of the primers is numbered according to the nucleotide sequence of BAC clone RP11-149I2 (GenBank accession: AL449423)

## Discussion

We have developed a general strategy that can be applied for pinpointing the genomic breakpoints in unpurified primary cancers. The amplification and tiling protocol described here allows for simple and precise *CDKN2A* breakpoint cloning, using contaminated DNA as a template. In contrast to the current available techniques for deletion mapping (including Southern blotting, fluorescent *in situ* hybridization, real time PCR, and array CGH) that rely on the absence of a detectable wild type signal, PAMP directly measures the deleted DNA. Therefore, this approach is much less vulnerable to problems associated with normal cell contamination. The experimental procedure is robust enough to detect deletions in the presence of at least 99.9% wild type sequence contamination, which could not be achieved by other procedures [3], [5], [26], [27], [28], [33], [35], [39].

Primer approximation PCR screening has been a useful tool for isolating deletion mutants in *C. elegans* [36]. The method relies on identifying a single band that is the product of a successful PCR reaction when a pair of specific primers is brought together by deletion, on an agarose gel. The procedure can only identify deletions that happen in a very small genomic fragment (3 kb) in a relatively low throughput fashion. It also suffers from relatively high false positive rate because the identity of the bands on the agarose gel is difficult to know. However, by applying multiplex PCR together with a genomic tiling array, one can simultaneously screen a wider range of genomic regions [40]. In addition, preferential amplification of the sequences near the breakpoints generates a relatively straightforward readout on the tiling array. The signal to noise ratio on the hybridized spots is obvious compared to the readout from array CGH (see Figure 4). The junction can be readily identified as long as one end of the nearby genomic location of the breakpoints is covered by the tiling array, as shown in the case of Hs578T breast cancer cell (Figure 6). Since high-density genomic tiling arrays are commercially available, this approach can be easily adopted. In addition, high-throughput genome sequencing technology may also pinpoint the exact breakpoint sequence after PAMP, bypassing the need for array hybridization [41], [42], [43].

We used multiplex PCR to reduce the workload and cost for PAMP. We were able to multiplex 28 primers easily in a single PCR reaction. Theoretically, one can cover over 90% of the 0.5 Mb of genomic fragment around *CDKN2A* locus with a total of 500 primers in one single PCR reaction through computational simulation, which will be described elsewhere (manuscript submitted). A recent paper reported a successful multiplex PCR with more than 1000 primer pairs through the aid of computational design [44]. The PAMP approach targets deletion sizes between 10 kb and 1 Mb. The smaller or larger deletions can be detected by resequencing and FISH respectively.

Like other PCR technologies, PAMP can be easily adopted to a robotic system for clinical and research purposes. One example of a potential clinical application is to use the unique breakpoint sequence as a personalized cancer-specific biomarker for disease monitoring after treatment, when the precise breakpoint has been mapped. For example, unlike many current tumor markers, such as CA19-9, CA125 and PSA, which are not truly cancer-specific, the *CDKN2A* breakpoints are specific and unique for each cancer with this locus deleted. A highly sensitive assay, such as real-time PCR, can be designed to monitor the status of cancer progression in the blood or other body fluids. The assay should be very specific because amplification is expected to occur only from deletion-containing DNA due to very long distance between the primers in the wild type genome (see Figure 2). This is analogous to the detection of a foreign virus sequence, which has been applied as a useful biomarker for Epstein-Barr virus associated nasopharyngeal carcinoma [45], [46].

Our approach can also ease traditional labor-intensive experiments that aim to understand how genomic breakpoints are generated during cancer development, particularly in primary tumors. Although illegitimate V(D)J recombination may be responsible for creating *CDKN2A* deletions in acute lymphoblastic leukemia, more breakpoint sequence data will be needed for other types of cancers to delineate the molecular mechanisms [37], [38], [47], [48]. Furthermore, the technique described in this paper can be used not only for deletion mapping, but it can also be applied to map other types of genomic rearrangement, such as translocations and inversions. Similar to the case of genomic deletion (see Figure 2), only “approximated” primers can generate amplicons when those primers are near the genomic fragments that are repositioned in translocations and inversions.

Using breakpoint sequences as cancer-specific biomarkers to monitor minimal residual diseases has been explored [49], [50] [51] [52] [53]. Disease monitoring based on personalized genomic DNA breakpoint is considered to be highly attractive approach for several reasons[49]. First, many genomic DNA rearrangements are directly related to oncogenic process, therefore, are truly cancer-specific and stable over time. This is in contrast to more convenient Ig/TCR rearrangement based assay. Indeed, we found exactly the same *CDKN2A* breakpoints of the two cell lines used in this study as reported by others. Second, the DNA is more stable than RNA although it is easier to map fusion transcript if it exists, such as *BCR-ABL*. Third, the genomic breakpoints are very likely to be different from each patient and become personalized biomarkers, thereby, reducing the risk of false positive results due to cross contamination. However, this is also the biggest hurdle to overcome. For example, many efforts to improve the PCR amplification range for detecting *C-MYC*/immunoglobulin translocations have had limited success because the breakpoints are scattered across a more than 300 kb region [54], [55], [56]. Our strategy may be useful for such application.

Our approach aims to identify breakpoints within a 1 Mb genomic fragment as FISH or other cytogenetic techniques are available for larger genomic rearrangements and the cost and labor significantly increase when the target region expands. We are able to inexpensively produce a tiling array covering a genomic fragment of 0.5 Mb around the *CDKN2A* with 1 kb resolution. We are currently working on methods to increase multiplexing and reduce the volume of each PAMP reaction for broader applications.

## Materials and Methods

### Cell culture and sample preparation

The cell lines described in the paper were obtained from the American Type Culture Collection (ATCC, Manassas, VA) and cultured as recommended. The genomic DNA was extracted with DNAzol (Molecular Research Center, Inc., Cincinnati, OH) following the instructions from the manufacturer.

### Minigenomic tiling array

We created an *INK4A* minigenomic tiling array covering a 25 kb fragment in the *CDKN2A* locus for proving the concept of our approach. DNA probes were generated by PCR with BAC clone RP11-149I2 (obtained from BACPAC Resources Center at Children's Hospital Oakland Research Institute, Oakland, CA) as template and avoiding the repetitive genomic sequences that were predicted by RepeatMasker. The PCR products were purified with DNA Clean-up and Concentrator-5 (Zymo Research, Orange, CA), resuspended in 3×SSC and printed on poly-L-lysine slides at 0.1 mg/ml along with Human Cot-1 DNA (Invitrogen, Carlsbad, CA), which is enriched for repetitive sequences, and herring sperm DNA (Promega, Madison, WI), which was used as nonspecific control. The printing procedure has been described and essentially followed the manual of the DeRisi arrayer with silicon microcontact printing pins (Parallel Synthesis Technologies, Inc. Santa Clara, CA) [57], [58]. Arrays were post-processed with succinic anhydride-based method for blocking before hybridization as previously described [57]. The protocols related to array printing and hybridization in this paper generally can be found in microarrays.org (http://derisilab.ucsf.edu/microarray/protocols.html).

### Primer-Approximation Multiplex PCR (PAMP) and array hybridization

A simplified PAMP scheme is shown in Figure 2. A series of primers (Table 2) toward *INK4A* exons 1-2 along the *CDKN2A* locus were synthesized by Integrated DNA Technologies (Coralville, IA). Groups of forward and reverse primers (250 nM each in the final reaction) were used to generate amplicons from 0.1 µg of genomic DNA templates in a total of 10 µl of solution mixing with 10 µl of Taq 2×Master Mix (New England Biolabs, Ipswich, MA). The reaction was assembled at 4°C in a PCR workstation and transferred to a thermocycler with the block preheated to 94°C. The cycling conditions were a 3-minute denaturation step at 94°C followed by 35 cycles at 92°C for 30 sec, 55°C for 30 sec and 68°C for 2.5 minutes with a final extension step at 68°C for 5 minutes. One µl of unpurified product was subsequently used as templates for another round of amplification to label the amplicons with the same PCR protocol except that dTTP was replaced by a 4∶1 mixture of aminoallyl dUTP (Ambion, Austin, TX) and dTTP for probe labeling. The labeled amplicons were purified with DNA Clean-up and Concentrator-5 columns, eluted in 9 µl of sodium bicarbonate (pH 9.0) and coupled with 1 µl of DMSO dissolved Cy3 or Cy5 NHS esters (GE Healthcare, Piscataway, NJ) for 30 to 60 minutes. The Cy3 and Cy5 labeled amplicons were purified with DNA Clean-up and Concentrator-5 columns and eluted with 10 µl of 10 mM Tris-HCl (pH 8.0). Paired Cy3 and Cy5 labeled amplicons were combined with 3.6 µl of 20×SSC, 0.5 µl of Hepes (pH 7.0) and finally 0.5 µl of 10% SDS. The mixed solution was heated for 2 minutes at 95°C, cooled to room temperature and hybridized to the minigenomic tiling arrays at 63°C overnight essentially as previously described [57], [58]. The hybridized arrays were washed and scanned with GenePix 4000B scanner (Molecular Device, Sunnyvale, CA) and analyzed by GenePix Pro 6.0 software.

### Tiling array data analysis

The human Cot-1 DNA and herring sperm DNA were designed to be positive and negative controls respectively and spotted multiple times on the array (see Figure 3). To normalize for day-to-day and sample-to-sample variation, the median intensity of all features representing herring sperm DNA (***I_50%-HS_***) were used to divide the intensity (***I_G_***) of each feature representing genomic probes. Each (***I_G_***):(***I_50%-HS_***) ratio, the normalized genomic probe signal, was plotted at the Y-axis against the corresponding probe's genomic location at the X-axis to ease data interpretation (see Figure 4).

### *CDKN2A* breakpoint cloning

To confirm *CDKN2A* breakpoint mapping by PAMP approach, the genomic fragments flanking the breakpoints were cloned by traditional PCR approaches guided by the results from PAMP. Two examples are given in this paper.

*Detroit 562 cell line*: The nested approach was used to clone the *CDKN2A* breakpoint in Detroit 562 human epithelial carcinoma cells. The primers were designed with clues from the PAMP experiment (see Figures 3 and 4). External primers (AGGTTTGGTTAAGAGTCGTTC and AAGATCTATATGGTGGCCTTTAG) were used for 35 cycles of PCR (92°C, 30 seconds; 55°C, 30 seconds; 68°C, 2 minutes). The two sets of internal primers (ATGCTAGCTGTAACTGGAGC and CTTAAGGCTAAATTGACTTG; GGCTTAGAGCTAACTCTTCACCC and TATGTGTGTGTGTGTCTGTGTGATG) were used for the second PCR reaction under the same conditions. A single band about 1 kb in size was excised and extracted with Qiaquick gel extraction kit (Qiagen, Valencia, CA). The purified product was directly sequenced with internal primers.

*Hs578T cell line*: Four sets of uniplex PCR reactions were performed by pairing 2 single forward (F_A1_ and F_A2_) and 2 single reverse (R_X3_ and R_X4_) primers. The PCR program was the same as that for PAMP. The products were analyzed by agarose gel electrophoresis. A single band from the shortest distance pair (F_A2_-R_X3_) was excised and sequenced with an internal primer.
